# Bone Marrow-Derived IL-1Ra Increases TNF Levels Poststroke

**DOI:** 10.3390/cells10040956

**Published:** 2021-04-20

**Authors:** Christian Ulrich von Linstow, Sofie Mozart Hindkjær, Pernille Vinther Nielsen, Matilda Degn, Kate Lykke Lambertsen, Bente Finsen, Bettina Hjelm Clausen

**Affiliations:** 1 Center for Neurodegenerative Science, Van Andel Institute, Grand Rapids, MI 49503, USA; ChristianLinstow@hotmail.com; 2 Department of Neurobiology Research, Institute of Molecular Medicine, University of Southern Denmark, 5000 Odense, Denmark; sofie.mozart@gmail.com (S.M.H.); pvnielsen@health.sdu.dk (P.V.N.); klambertsen@health.sdu.dk (K.L.L.); bfinsen@health.sdu.dk (B.F.); 3 Department of Pediatrics and Adolescent Medicine, University Hospital Rigshospitalet, 2100 Copenhagen, Denmark; matildadegn@gmail.com; 4 Department of Neurology, Odense University Hospital, 5000 Odense, Denmark; 5 BRIDGE—Brain Research—Inter-Disciplinary Guided Excellence, Department of Clinical Research, University of Southern Denmark, 5000 Odense, Denmark

**Keywords:** mice, brain, stroke-related genes, inflammation, cell therapy, microglia, cytokines, chemokines, serum

## Abstract

Tumor necrosis factor (TNF) and interleukin-1 receptor antagonist (IL-1Ra) are key players in stroke, a disease in which cell-based therapies have shown great potential. Having shown an infarct-reducing effect of bone marrow (BM) cells, especially cells with high IL-1Ra expression, we here investigated the effect of BM cells on TNF and other stroke-related mediators in mice after transient middle cerebral artery occlusion (tMCAo) and in vitro using adult microglial cultures. We analyzed stroke-related genes and inflammatory mediators using qPCR stroke Tier panels, electrochemiluminescence, or enzyme-linked immunosorbent assays. We found a significant correlation and cellular colocalization between microglial-derived TNF and IL-1Ra, though IL-1Ra production was TNF independent. BM treatment significantly increased TNF, interleukin (IL)-10, and IL-4 levels, while C-X-C motif ligand 1 (CXCL1), IL-12p70, and Toll-like receptor 2 (TLR2) decreased, suggesting that BM treatment favors an anti-inflammatory environment. Hierarchical clustering identified *Tnf* and *IL-1rn* within the same gene cluster, and subsequent STRING analysis identified TLR2 as a shared receptor. Although IL-1Ra producing BM cells specifically modulated TNF levels, this was TLR2 independent. These results demonstrate BM cells as modulators of poststroke inflammation with beneficial effects on poststroke outcomes and place TNF and IL-1Ra as key players of the defense response after tMCAo.

## 1. Introduction

Cellular therapy has emerged as an experimental treatment strategy for stroke, and accumulating evidence supports the role of stem cells as modulators of stroke-induced inflammation [1]. Following ischemic stroke, microglial activation is widespread. Peri-lesional microglia and blood-borne infiltrating leukocytes directly impact the survival of injured neurons within the penumbra [2,3,4,5,6,7,8,9]. Microglia and leukocytes release inflammatory mediators with partially overlapping but also opposing effects on cerebral infarction and poststroke outcome [8,10,11,12]. The strength of cellular therapies in stroke relies on the ability of stem cells or bone marrow (BM) to infiltrate the penumbra, thereby allowing them to modulate detrimental responses directly [11,13,14,15,16] and stimulating repair mechanisms in situ [17,18,19].

Tumor necrosis factor (TNF) is a pleiotropic cytokine known to participate in acute stroke injury and delayed stroke recovery [10,20]. TNF is synthesized as a monomeric transmembrane protein that assembles into homotrimeric membrane TNF. Following enzymatic cleavage by the matrix metalloprotease ADAM-17, also known as TNF-alpha converting enzyme (TACE), membrane TNF is converted into soluble TNF [21]. TNF mediates its biological activity through the two TNF receptors, TNFR1 and TNFR2, which have different affinities for membrane and soluble TNF, respectively [22,23]. The current view is that signaling by soluble TNF through TNFR1 increases the infarct volume (IFV), whereas signaling by membrane TNF through the TNFR2 is neuroprotective [12]. TNF is upregulated early after an ischemic insult [8,20,24], particularly in activated brain resident microglia and infiltrating leukocytes located to the penumbra in humans and rodents [8,20,24,25].

We have previously shown that treatment with BM cells induces the production of interleukin (IL)-1 receptor antagonist (IL-1Ra) in resident microglia, an effect also associated with a reduced IFV and improved functional outcomes after middle cerebral artery occlusion (MCAo) in mice [11]. The signals that led to the increased IL-1Ra production in microglia were not investigated in our original manuscript, but studies have shown that TNF in synergy with IL-10 and IL-4 can increase the production of IL-1Ra in activated human polymorphonuclear cells (PMNs) [26]. Local inflammation induced by TNF has also been suggested to mobilize endogenous bone marrow (BM) cells to the site of injury [27], and it is known that TNF is upregulated in the ischemic penumbra early after stroke onset [24]. Here, we investigated how BM treatment, which amplifies the production of IL-1Ra in the resident microglia, affects microglial TNF response after MCAo in mice. To facilitate cellular infiltration, the study was performed in mice subjected to transient MCAo (tMCAo), followed by BM treatment 30 min (min) after reperfusion. We demonstrate that after tMCAo, BM cells increase the tissue levels of TNF, IL-10, and IL-4 and downregulates CXCL1 and IL-12p70 without affecting IL-6. IL-1Ra producing BM cells specifically modulate brain TNF levels, and both link to the defense response poststroke. The results show a strong correlation between TNF and IL-1Ra levels and that these two cytokines are produced by largely the same subset of microglia in the brain parenchyma poststroke.

## 2. Materials and Methods

### 2.1. Study Design and Animal Ethics

**Figure 1:**
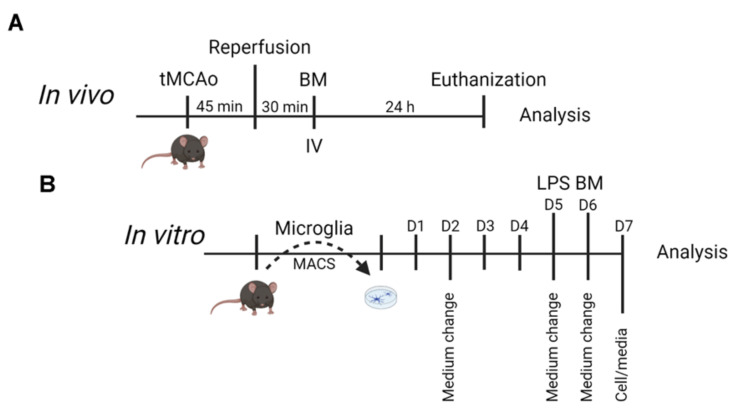
Schematic illustration of the experimental timeline. The timeline of the in vivo experiment is shown in (**A**) and the in vitro experiments in (**B**). BM; bone marrow, LPS, lipopolysaccharide, MACS; magnetic assisted cell sorting; tMCAO, transient middle cerebral artery occlusion. (the experimental overview was created with BioRender.com, accessed on 8 April 2021).

In order to investigate if BM with elevated and normal expression of IL1-Ra affects microglial TNF response after tMCAo, we used brain material (tissue sections and serum) from the BM-treated tMCAo mice generated in Clausen et al. [11] (Figure 1A). To obtain more mechanistic information, we also studied cultured adult microglia from C57BL/6 mice and mice with knockdown of TNF in microglia and littermate (LM) controls (Figure 1B). Mice were housed in the Laboratory of Biomedicine, University of Southern Denmark. Data are reported in accordance with the ARRIVE (Animal Research: Reporting of In Vivo Experiments) guidelines. Samples from mice subjected to tMCAo with the inclusion of unlesioned controls were selected randomly and blinded to the principal investigator. Blinding was lifted after data analysis. No inclusion or exclusion criteria were set prior to analysis, and no data have been excluded from the dataset. Animal experiments followed the guidelines of the Danish Animal Inspectorate, and all efforts were made to minimize pain and distress (J. No. 2011/561-1950).

### 2.2. Mice Used for tMCAo and BM Treatment

This study was conducted on adult male IL-1Ra transgenic (Tg) mice (hemizygous mice carrying the transgene encoding secreted IL-1Ra mRNA under the control of its endogenous promoter) and LM controls [11,28]. Transient (t)MCAo was performed under 1–2% isoflurane anesthesia. Mice received 1 mL (s.c.) 0.9% saline before the transient intraluminal filament technique (45 min) [29]. Blood flow and body temperature were monitored using an optical fiber probe (T6a and VP10 M200st, Moor Instruments, UK) connected to a laser Doppler flowmeter (Moor Instruments, UK). Postsurgery, the mice received 0.1 mL (s.c.) 5% glucose and 0.1 mL Temgesic (buprenorphium 0.3 mg/mL; pharmaceuticals, USA). Temgesic was given at 8 h intervals for the first 24 h poststroke [11].

BM harvesting, characterization, and treatment have previously been detailed [8,11]. Approximately 1 × 10^7^ BM cells were injected into the tail vein of recipient mice 30 min after tMCAo [11]. In this study, we include samples from LM mice subjected to tMCAo (LM) (*n* = 10), LM mice subjected to tMCAo, followed by LM BM treatment (LM–LM) (*n* = 12), or IL-1Ra BM treatment (Tg–LM mice) (*n* = 12), and unlesioned controls (Ctl) (*n* = 11) [11].

### 2.3. Mice Used for Adult Microglial Cultures

Adult microglial cultures were prepared from adult female C57BL/6JBomTac (Taconic A7S, Denmark), *Cx3cr1^CreER^Tnf^fl/fl^* with Tamoxifen-induced knockdown of TNF in microglia, and *Tnf^fl/fl^* LM control mice [30]. In *Cx3cr1^CreER^Tnf^fl/fl^* mice, Cre recombination of floxed alleles was induced by tamoxifen injections for five consecutive days (0.1 mg/day injected intraperitoneally (IP)), followed by a 28-days waiting period [31]. *Tnf^fl/fl^* LM mice received the same treatment. In addition, B6.NZeg transgenic mice, in which enhanced green fluorescent protein (EGFP) was fused to a nuclear localization signal ubiquitously expressed from the CAG promoter, were used as a source of GFP^+^ BM cells. A total of 5 × 10^5^ BM cells, harvested from either B6.NZeg, C57BL/6, or IL-1Ra-Tg mice, were used for BM treatment in cultured microglia.

### 2.4. Tissue Processing

Brain tissue was snap frozen in gaseous CO_2_ and processed into six parallel series of 30 μm thick cryostat sections [11]. For this study, we used tissue sections collected in Eppendorf tubes for qPCR (Tier 1 and Tier 2 analysis) and one series of sections for electrochemiluminescence analysis and enzyme-linked immunosorbent assay (ELISA) for cytokines, chemokines, and TLR2. As described, parallel series of sections have previously been used for volumetric infarct analysis, as well as mRNA and protein analysis of IL-1 [11]. Paraformaldehyde fixed brain tissue was processed into 12 parallel series of sections (16 μm), as previously detailed [8].

### 2.5. Electrochemiluminescence and ELISA Analyses

For protein determination, one series of brain tissue was sonicated in cold phosphate-buffered saline (PBS) (Sigma life science, St. Louis, MO, USA) containing phosphatase inhibitors (Sigma-Aldrich, Soeborg, DK) and cOmplete mini, EDTA-free proteinase inhibitor cocktail (Roche, Basel, Switzerland). The total protein content was measured using the bicinchoninic acid (BCA) assay according to the manufacturer’s protocol (P4417-100TAB, Thermo Scientific, Waltham, MA, USA).

Electrochemiluminescence analysis was performed on brain, serum, cells, and media using the MSD Mouse Proinflammatory V-Plex Plus Kit (IFNγ, IL-2, IL-4, IL-5, IL-6, IL-10, IL-12p70, CXCL1, TNF; K15012C, Mesoscale Discovery), the U-Plex Kit (TNFR1; K15069L, Mesoscale), and the R-Plex kit (TNFR2; K150ZSR-2, Mesoscale Discovery) according to the manufacturer’s instructions. Plates were read using the MSD QuickPlex (SQ120) Plate Reader (Mesoscale Discovery), and the data analyzed using the MSD Discovery Workbench software as detailed [20].

IL-1Ra ELISA was performed as specified in the Quantikine mouse IL-1Ra assay (MRA00, R&D). TLR2 ELISA was performed as specified in the mouse TLR2 Elisa kit (ab224880, Abcam). The measured protein was normalized to the total protein content measured using the BCA assay [32].

### 2.6. Quantitative PrimePCR

RNA extraction and cDNA synthesis were performed as previously detailed [33], and qPCR was performed using predesigned 96-well PrimePCR disease panels (Stroke Tier 1 panel and Stroke Tier 2 panel, Bio-Rad, Copenhagen, DK) with SYBR Green. A positive PCR control assay, DNA contamination control assay, RNA quality control assay, and reverse transcription control assay were included and further detailed in the manufacturer’s protocol (Bio-Rad, DK). The qPCR was performed at StepOnePlus (Thermo Fisher Scientific, Odense, DK). The PrimePCR disease state panels with a total of 187 differentially expressed genes were designed referencing the National Library of Medicine database (www.nlm.nih.gov, accessed on 9 April 2021). Tier 1 plates represented higher ranked targets, whereas Tier 2 plates contained the lower-ranked or less well-characterized targets.

Following each qPCR run, the Cq values of each plate were copied into the Stroke Tier 1 or Stroke Tier 2 run files (downloaded from www.bio-rad.com) and saved. Next, the brain PCR run files were imported into the PrimePCR data analysis software (www.bio-rad.com/PrimePCR and analyzed). The gene study was created by importing all brain PCR run files (both for Tier 1 and Tier 2, 17 plates/each) into the PrimePCR analysis software. First, all plates were normalized to a control plate, which contained brain cDNA pooled from 10 unlesioned C57BL/6 male mice, and then inter-run calibrated to the reference genes hypoxanthine phosphoribosyltransferase 1 (*Hprt*), glyceraldehyde 3-phosphate dehydrogenase (*Gapdh*), and Histone H3.3 (*H3f3a*) before further analysis.

### 2.7. Adult Microglia Cultures

Microglial cell cultures were generated from cortical microglial cells freshly harvested from adult female C57BL/6 mice and tamoxifen-treated *Cx3cr1^CreER^Tnf^fl/fl^* or littermate *Tnf^fl/fl^* mice [30]. In brief, mice were euthanized by cervical dislocation, the meninges were removed, and the brains were quickly collected for microdissection. The cortices were placed in ice-cold Hank’s balanced salt solution (HBSS) (Gibco) and centrifuged at 300× *g* for 5 min, at room temperature (RT), followed by 5 min in cold Dulbecco’s PBS (DPBS) (Gibco Laboratories, Gaithersburg, MD, USA). The cortices were dissociated using the Neural Tissue Dissociation Kit (Miltenyi Biotec, Bergisch Gladbach, Germany) and the gentleMACs Octo Dissociator (Miltenyi Biotec), as detailed in the manufacturer’s guidelines. Cell homogenates were centrifuged, and the pellet resuspended in 10 mL cold DPBS and filtered through a sterile 70 µm smart strainer (Miltenyi Biotec). Cell numbers were counted using a Bürker-Türk counting chamber. Next, the cell homogenates were centrifuged at 300× *g* for 10 min at RT. For the positive selection, cells were resuspended in 0.5% bovine serum albumin (BSA) in DPBS, containing 10 µL CD11b microbeads (Miltenyi Biotec) per 1 × 10^7^ cells for 15 min at 4 °C. Next, cells were washed in 0.5% BSA in DPBS and loaded onto the LS columns (Miltenyi Biotec). CD11b^+^ cells were isolated using 3 mL 20% fetal bovine serum (FBS) in Dulbecco’s modified eagle’s medium (DMEM) (Thermo Fisher), heat-inactivated FBS, penicillin–streptomycin, minimum essential medium (MEM), nonessential amino acid solution, pyruvate, and GlutaMAX (Gibco Laboratories, Gaithersburg) Collected, CD11b^+^ cells were plated onto a 24-well poly-l-lysine (PLL)-coated plate (10 μg/mL PLL in DPBS) with approximately 200,000 cells per well for protein analysis (~1000 cells/mm^2^) and approximately 75,000 cells per well for immunofluorescence staining (~400 cells/mm^2^). Cell cultures were incubated in controlled atmospheric conditions of 5% CO_2_ and 95% humidified air at 37 °C. Conditioned media was changed on days 2, 5, and 6, and cultures were harvested for protein analysis or fixed in 4% paraformaldehyde (PFA) on day 7. Experimental groups consisted of 1) unstimulated microglia (Ctl), 2) lipopolysaccharide (LPS) stimulated microglia (LPS^+^ Mic), 3) BM-treated (5000 cells/mm^2^) microglia (BM^+^ Mic) (total cell density ~3700 cells/mm^2^), and 4) LPS stimulated + BM treated microglia cultures (LPS^+^BM^+^ Mic). LPS (from *Escherichia coli* 0111:B4, Sigma-Aldrich, St. Louis, MO, USA) stimulation was performed on day 5. LPS (100 ng/mL) [34,35] was added to each well in freshly made preheated 10% FBS–DMEM medium. By day 6, the cells were washed and reincubated with freshly isolated BM cells in preheated 10% FBS–DMEM medium. A total of 26 C57BL/6JBomTac mice and nine tamoxifen-treated mice (*Cx3cr1^CreER^Tnf^fl/fl^* and *Tnf^fl/fl^* LM control mice) were used in this study, amounting to 3–5 wells/experimental condition across independent experiments to account for methodological and biological bias.

### 2.8. Immunofluorescence

Microglia cultures used for immunofluorescent staining were incubated in ice-cold DMEM for 5 min before fixed in 4% paraformaldehyde (PFA) in 0.15 M Sorenson’s buffer for 20 min at RT. Next, cells were washed twice with 0.15 M Sorenson’s buffer, and 500 μL 0.15 M Sorenson’s buffer was added to each well before stored at 4 °C. Immunofluorescence (double- or triple fluorescence) stainings were performed on tissue sections from stroke lesioned mice and microglial cell cultures, as previously described [8]. First, tissue sections or cells were rinsed in tris-buffered saline (TBS) containing 0.5% Triton for 10 min at RT. Next, tissue sections or cells were blocked with 10% FBS in TBS for 30 min, followed by incubation with the primary antibody in 10% FBS in TBS overnight (O.N) at 4 °C. The next day, sections or cells were rinsed before incubated with a species–specific secondary fluorescent antibody for 1–2 h. While protected from light, the sections or cells were next rinsed in TBS, distilled H_2_O, and mounted in ProLong Gold antifade reagent with 4′,6-diamidino-2-phenylindole (DAPI; Invitrogen, Carlsbad, CA, USA). The specificity of the stainings (Primary antibodies: TNF (P-350, Endogen), IL-1Ra (AF-480-NA, R&D systems); CD45-PE (Clone 30-F11, BD Biosciences), CD11b (MCA711, Serotec) or CD68 (MCA1957GA, Serotec); secondary antibodies: 594-conjugated anti-rabbit antibody (A21207, Invitrogen), 488- or 594-conjugated anti-goat antibody (A21467, Invitrogen), or 488-conjugated anti-rat antibody (A11006, A21208, Invitrogen) or Cy5-conjugated anti-rat (AB_2340672, Jackson ImmunoResearch). Antibody specificity was verified by substituting the primary antibody with the respective isotype (IgG) (X0903, DakoCytomation; Clone A95-1, BD Biosciences; Clone RTK4530, BioLegend) or serum (Ig) controls (X0907, DakoCytomation). Control stainings were devoid of signal.

### 2.9. Statistics

Quantitative data are presented as mean ± SEM. Comparison between two groups was performed using Student’s *t*-test. Multiple comparisons were performed using one-way ANOVA followed by Sidak’s post hoc tests. Correlations were established using the parametric Pearson test. Statistical analysis was performed using the Prism 5 software for Windows (GraphPad). Statistical significance was established for *p* < 0.05.

## 3. Results

### 3.1. TNF and IL-1Ra Transcript Levels Are Affected by tMCAo in Mice

**Figure 2:**
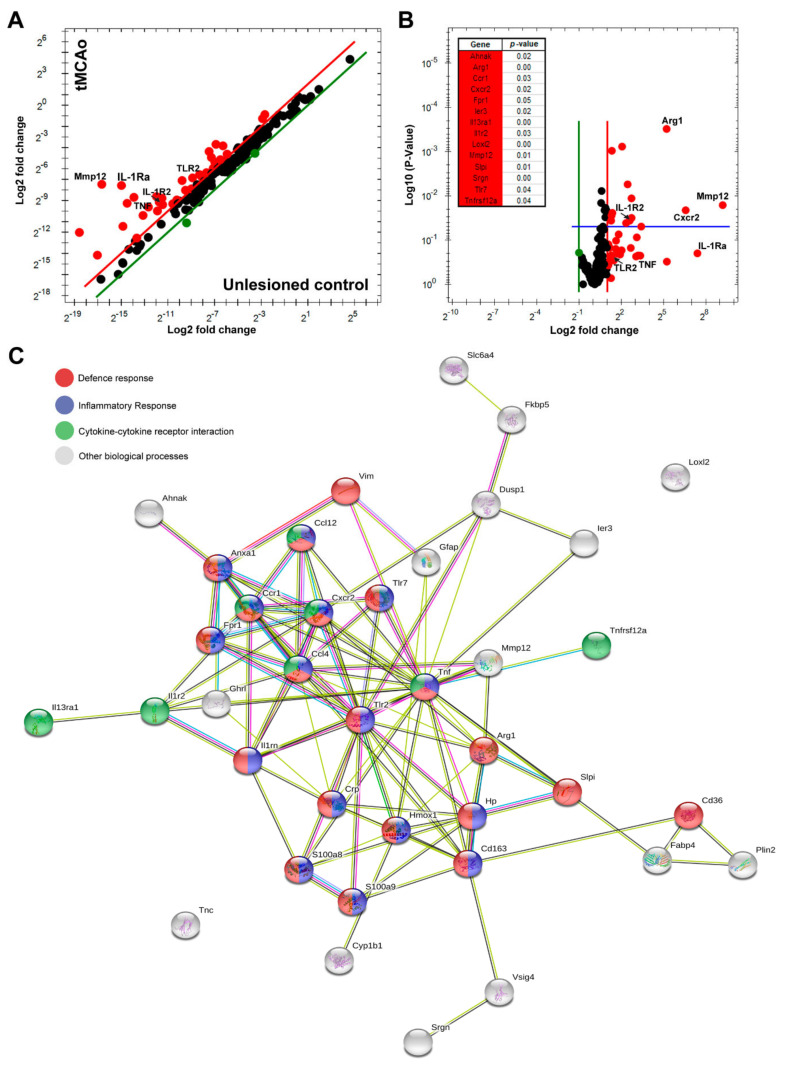
Tumor necrosis factor (TNF) gene expression is increased after tMCAo in mice. (**A**) Scatter and (**B**) volcano plot showing log2 Fc in 38 out of 187 stroke-related genes identified by qPCR. The volcano plot shows the *p*-values for the identified genes with biological and statistical significance (*p* < 0.05, above the blue line). Upregulated gene transcripts beyond a twofold change are depicted in red color and downregulated transcripts in green color. (**C**) Gene network of the 38 genes identified. STRING interaction network depicting genes involved in the defense response (red nodes), the inflammatory response (blue nodes), and cytokine–cytokine receptor interaction (green nodes). White nodes represent genes involved in other biological processes.

To obtain an overview of stroke-induced changes in transcript levels, we used PrimePCR disease state panels Stroke Tier 1 and 2. This allowed us to analyze 187 differentially expressed genes involved in stroke. The analysis identified 38 genes with log2 fold changes (Fc) in transcript levels (Figure 2A,B), showing the genes with *p*-values < 0.5 (Figure 2B) 24 h after tMCAo compared to unlesioned control mice. STRING network analysis was performed to reveal protein–protein interaction networks between the identified genes (Figure 2C). Based on interaction evidence [36], genes involved in the defense response, the inflammatory response, and cytokine–cytokine receptor interaction 24 h after tMCAo were highlighted (Figure 2C). *Tnf* was identified in all three pathways, whereas *IL-1rn*, the transcript giving rise to IL-1Ra, was identified in the defense and the inflammatory response 24 h after tMCAo (Figure 2B,C). Interestingly, *Tlr2* appeared to be a shared link between *Tnf* and *IL-1rn* (Figure 2C).

### 3.2. IL-1Ra BM Treatment Increases TNF Protein Levels after tMCAo

**Figure 3:**
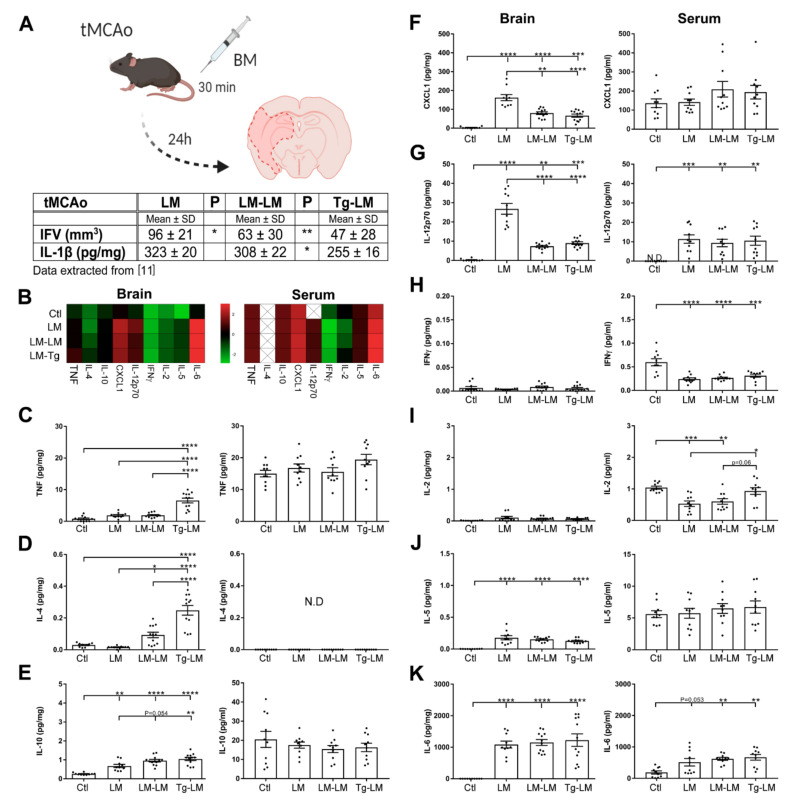
Effect of bone marrow (BM) cells on ischemia-induced proteins in brain and serum. (**A**) BM cell treatment 30 min after tMCAo reduces infarct volumes (IFV) in LM–LM and Tg–LM mice as well as brain IL-1β levels (pg/mg) in Tg–LM mice 24 h after tMCAo, compared to LM tMCAo mice (Table with extracted data from [11]) (the experimental overview was created with BioRender.com). (**B**–**K**) Comparison of TNF (**C**), IL-4 (**D**), IL-10 (**E**), CXCL1 (**F**), IL-12p70 (**G**), IL-5 (**H**), IL-6 (**I**), IFNγ (**J**) and IL-2 (**K**), levels in the brain (pg/mg) and serum (pg/mL) of unlesioned controls and in LM, LM–LM, and Tg–LM mice 24 h after tMCAo. Data are presented as mean ± SEM (*n* = 10–12/group). One-way ANOVA, followed by Sidak’s post hoc tests, was performed. Significance indicated as * *p* < 0.05, ** *p* < 0.01, *** *p* < 0.001, **** *p* < 0.0001. N.D, not detected.

TNF is known to have both beneficial and deleterious effects in stroke, whereas IL-1Ra selectively antagonizes the deleterious effect of IL-1 [10]. We have previously shown that BM cells with high IL-1Ra expression reduce IFV and decrease IL-1β protein levels in the brain 24 h after permanent but also tMCAo (Figure 3A, data extracted from [11]. However, whether BM cells with high IL-1Ra expression also affect TNF expression was not investigated in our former study. Here, we, therefore, studied the protein expression of TNF and a panel of inflammatory markers (e.g., IL-4, IL-10, chemokine (C-X-C motif) ligand 1 (CXCL1), IL-12p70, interferon (IFN)γ, IL-2, IL-5, and IL-6 in the brain and serum of tMCAo LM mice, LM–LM mice, Tg–LM mice, and unlesioned controls (Ctl) (Figure 3B–K). We found that BM cells from IL-1Ra Tg mice significantly increased brain TNF, IL-4, and IL-10 levels, compared to BM cells from LM mice 24 h after tMCAo and compared to unlesioned controls (Figure 3B–E). Both BM treatments significantly lowered brain CXCL1 and IL-12p70 levels compared to mice subjected to tMCAo alone (Figure 3F,G). Overall, we found no changes in levels of IFNγ or IL-2 in the brain (Figure 3H,I). IL-5 and IL-6 were significantly elevated in the brains of tMCAo LM, LM–LM, and Tg–LM compared to unlesioned controls (Figure 3J,K). Transient MCAo and BM treatment did not change serum TNF, IL-4, IL-10, CXCL1, or IL-5 levels (Figure 3B–F,H). Elevated levels of IL-12p70 and IL-6, however, were observed in the serum of mice 24 h after tMCAo and in LM–LM and Tg–LM-treated mice (Figure 3G,I). Overall, IFNγ was reduced in serum 24 h after tMCAo compared to unlesioned control mice (Figure 3J). We observed reduced levels of IL-2 in the serum of mice subjected to tMCAo, and mice subjected to tMCAo receiving BM cells from LM mice (LM–LM), however, not in serum from mice subjected to tMCAo receiving BM cells from IL-1Ra-overproducing mice (Tg–LM) (Figure 3K).

### 3.3. IL-1Ra BM Treatment Is Associated with a Unique Set of Transcripts after tMCAo

Next, we studied the effect of BM treatment on poststroke gene transcript expression using the PrimePCR disease state panels stroke Tier 1 and 2 in parallel brain samples from the same mice as used for protein analysis. First, we compared LM–LM and Tg–LM with unlesioned control mice to strengthen further and verify genes affected by tMCAo alone. As observed in the LM mice (Figure 2), genes such as *Tnf, IL-1rn, Tlr2, Il1r2, Ccl4, Ccl12, Ccr1, Cxcr2, Crp, Fprl,* and *Hmox1*, which are involved in the defense and inflammatory response, were identified in both LM, LM–LM, and Tg–LM mice 24 h after tMCAo compared to unlesioned control mice (Supplemental Figure S1A,B, and Supplementary Table S1).

**Figure 4:**
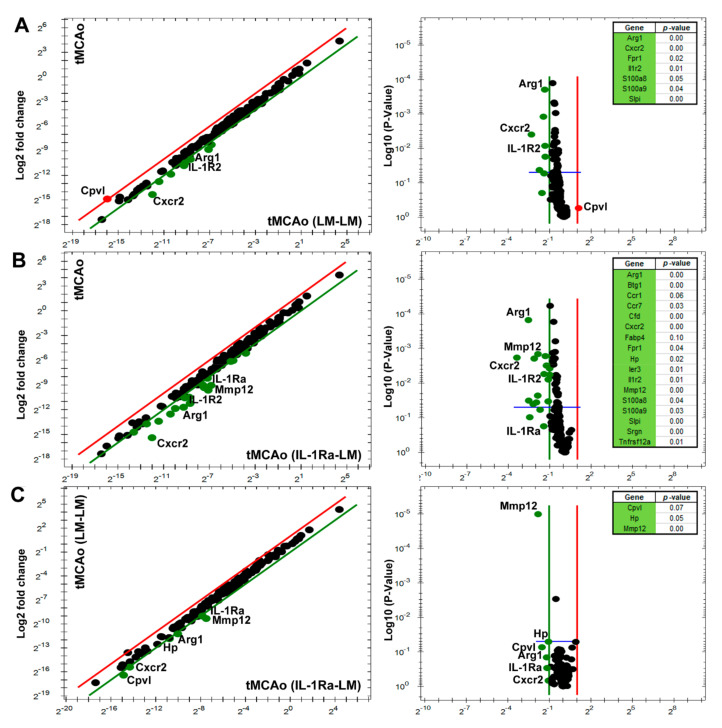
Gene expression affected by poststroke BM treatment in mice. (**A**–**B**) Scatter and volcano plots showing gene changes in LM–LM treated (**A**) and Tg–LM treated mice (**B**) 24 h after tMCAo, compared to tMCAo mice (*n* = 5 mice) without BM treatment. (**C**) Scatter plot and volcano plot showing gene changes in LM–LM treated mice, compared to Tg–LM treated mice 24 h after tMCAo. Upregulated gene transcripts beyond a log2 Fc are depicted in red color and down-regulated transcripts in green color. The volcano plot shows the *p*-values for the identified genes with biological and statistical significance (*p* < 0.05, blue line).

The comparison of LM–LM-treated mice to LM mice 24 h after tMCAo, identified a log2 Fc change in genes such as *Arg1*, *Cxcr2*, *Fabp4*, *Fpr1*, *Il1r2*, *S100a8*, *S100a9*, *Slpi*, and *Cpvl*, again highlighting genes with *p*-values < 0.5 (Figure 4A and Supplementary Table S1). However, when we compared Tg–LM mice to LM mice 24 h after tMCAo, we identified a Log2 Fc change in additional genes such as *Btg1*, *Ccr1*, *Ccr7*, *Cfd*, *Hp*, *Ler3*, *Il1rn*, *Mmp12*, *Srgn,* and *Tnfrsf12a,* highlighting the effect of IL-1Ra producing BM cells and genes with *p*-values < 0.5 (Figure 4B and Supplementary Table S1).

Direct comparison between LM–LM and Tg–LM treated mice 24 h after tMCAo identified a Log2 Fc change in genes such as *Arg1*, *Cpvl*, *Cxcr2*, *Hp*, *IL-1rn*, and *Mmp12*, with few genes showing *p*-values < 0.05 (Figure 4C and Supplementary Table S1). *Cpvl* was upregulated in LM–LM treated mice but not in Tg–LM treated mice.

**Figure 5:**
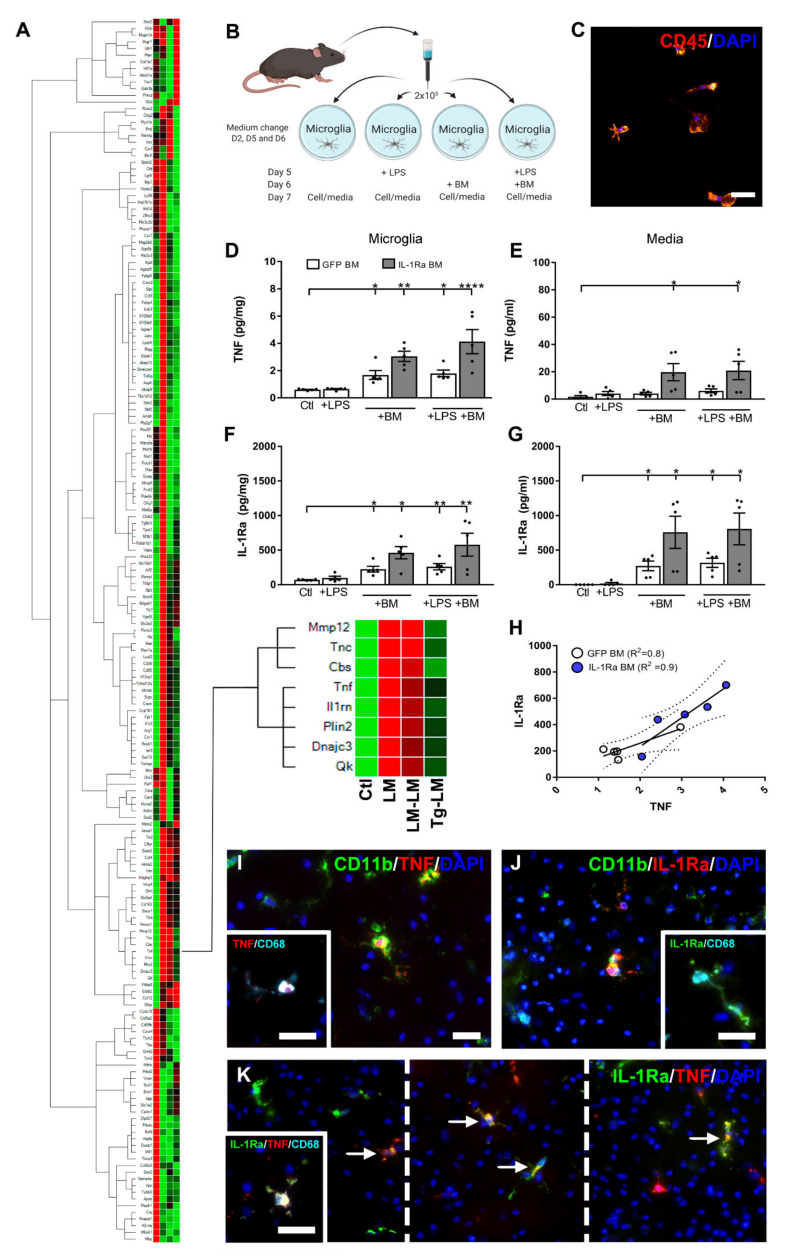
BM cells affect microglia TNF and IL-1Ra expression in tMCAo mice. (**A**) Hierarchical clustergram showing the degree of similarities in relative gene expression for different targets and samples showing, e.g., tMCAo LM mice, LM–LM mice, Tg–LM mice, and unlesioned controls (*n* = 5–11 mice/group). (**B**) Illustration of the experimental microglia culture setup. MACS sorted CD11b^+^ microglia from C57BL/6 mice were plated, stimulated with LPS on day 5 (D5), and treated with BM cells on day 6 (D6), or both, and cells and media were harvested on day 7 (D7). Media was changed on D2, D5, and D6 (the experimental overview was created with BioRender.com). (**C**) Fluorescent staining of CD45^+^ microglia D7 after culturing. (**D**,**E**) Protein analysis of TNF (**D**,**E**) and IL-1Ra (**F**,**G**) in microglia and media D7 after culturing (**D**–**G**). (**H**) Correlation analysis between TNF and IL-1Ra in cultured microglial cells treated with GFP BM or IL-1Ra BM cells. (**I**) Fluorescence staining of CD11b^+^ and CD68^+^ microglia coexpressing TNF 24 h after tMCAo in mice. (**J**) Fluorescent staining of CD11b^+^ and CD68^+^ microglia coexpressing IL-1Ra 24 h after tMCAo in mice. (**K**) Fluorescent staining of TNF^+^ microglia coexpressing IL-1Ra 24 h after tMCAo in mice, including the microglia marker CD68. Cells localize to the border of the ischemic infarct. Statistical data are presented as mean ± SEM (5 experimental cultures/group). One-way ANOVA, followed by Sidak’s post hoc tests, was performed. Significance indicated as * *p* < 0.05, ** *p* < 0.01, *** *p* < 0.001, **** *p* < 0.0001 (**B**). Scale bar: 10 μm (**G**–**I**).

Interestingly, hierarchical clustering analysis of all genes analyzed in LM, LM–LM, and Tg–LM, including unlesioned controls, placed *Tnf* and *IL1rn* in the same cluster 24 h after tMCAo (Figure 5A).

### 3.4. BM Cells Increase Microglial TNF Expression and Secretion

Changes in TNF and IL-1Ra have been shown to impact infarct development [11,12,24,37]; however, a potential common link between the two cytokines has, to our knowledge, not previously been described. Since microglia are the primary producers of both TNF and IL-1Ra poststroke [8,11], we next investigated the impact of BM cells on the microglia in vitro. We prepared microglia cell cultures from adult C57BL/6 mice and stimulated the microglia with LPS on day 5, BM cells on day 6 (either GFP or IL1Ra BM cells), or a combination of LPS and BM cells (Figure 5B). On day 7, CD45+ microglia (Figure 5C) were collected and processed for TNF and IL-1Ra protein analysis (Figure 5D,E). By day 7, TNF was significantly elevated in microglia treated with either GFP or IL-1Ra BM cells, compared to control microglia, with a similar tendency observed in LPS BM microglia cultures (Figure 5D). Interestingly, we observed that the IL-1Ra BM cells were more prone to increase TNF, compared to GFP BM cells (Figure 5D), which is a result that supports in vivo findings showing elevated TNF in Tg–LM treated mice poststroke (Figure 3C). In the culture media, we also observed an increase in TNF when the microglia were treated with IL-1Ra+ BM cells but not when treated with GFP BM cells (Figure 5E). LPS stimulation on day 5 did not affect TNF production in LPS+ microglia nor LPS+ BM microglia or media on day 7 (Figure 5D,E: white bars), which is most likely a result of the media shift on day 6. Analysis of IL-1Ra showed elevated levels of IL-1Ra in microglia treated with both GFP and IL-1Ra BM cells, compared to microglia controls, with a similar tendency observed in LPS+ microglia (Figure 5F). As expected, the use of IL-1Ra BM cells significantly increased IL-1Ra levels, compared to GFP BM cells, as seen both in BM microglia and LPS+ BM microglia (Figure 5F). In the culture media, we observed increased levels of IL-1Ra when microglia were treated with either GFP or IL-1Ra BM cells, compared to microglial controls (Figure 5G). Again, LPS stimulation on day 5 did not affect IL-1Ra production in LPS+ microglia nor LPS+ BM microglia or media on day 7 (Figure 5F,G: white bars). Higher levels of IL-1Ra were measured in the media from microglia treated with IL-1Ra BM cells, compared to GFP BM cells (*p* = 0.08) (Figure 5G).

Since *Tnf* and *IL-1rn* genes were found to cluster together in mice treated with BM cells 30 min after tMCAo (Figure 5A), we decided to perform correlation analysis between TNF and IL-1Ra proteins. We found a positive correlation between TNF and IL-1Ra in microglia treated with GFP BM cells (Pearson R2 = 0.8, *p* < 0.05) (Figure 5H) and IL-1Ra BM cells (not shown, Pearson R2 = 0.9, *p* < 0.03), including media from both cell cultures (not shown, GFP BM cells: Pearson R2 = 0.9, *p* < 0.04; IL-1Ra+ BM cells: Pearson R2 = 0.8, *p* < 0.03). These findings were further supported by a positive correlation between TNF and IL-1Ra protein levels in LM mice following tMCAo (not shown, Pearson R2 = 0.9, *p* < 0.008).

Due to these findings, we next investigated whether TNF and IL-1Ra, which are expressed by CD11b^+^ and CD68^+^ microglia (Figure 5G,H), could be expressed by the same microglia in vivo after MCAo. Double-immunofluorescence staining showed that TNF and IL-1Ra were largely expressed by the same microglia but with subsets of cells located in the ischemic penumbra also expressing either TNF or IL-1Ra alone (Figure 5I). Triple-immunofluorescence staining confirmed TNF or IL-1Ra to be expressed by CD68^+^ microglia (Figure 5I, insert). These findings support our cluster analysis indicating that elevated transcript levels of TNF and IL-1Ra mRNA could be induced by a shared stimulus in subsets of microglia after MCAo in mice.

### 3.5. TNF Does Not Affect IL-1Ra Production in Microglia

**Figure 6:**
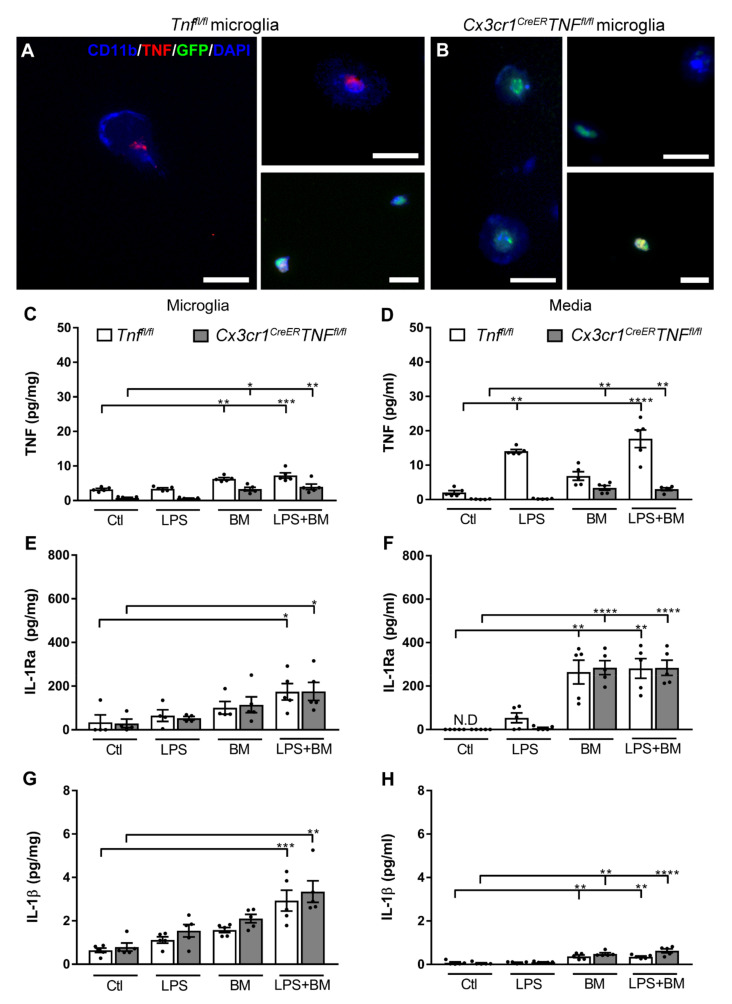
Effect of TNF on IL-1Ra expression. (**A**,**B**) Fluorescence staining showing coexpression between CD11b^+^ or GFP^+^ cells and TNF in microglia from *Tnf^fl/fl^* (**A**) and *Cx3cr1^CreER^Tnf^fl/fl^* mice (**B**). Stainings were performed on all cultures but only shown for microglia treated with LPS and GFP^+^ BM cells (**A**,**B**). (**C**–**F**) CD11b MACS sorted microglia from tamoxifen-treated *Tnf^fl/fl^,* and *Cx3cr1^CreER^Tnf^fl/fl^* mice were stimulated with LPS (D5), GFP BM cells (D6), or both. Microglia and media were harvested on D7 and analyzed. Microglia-conditioned media was changed on D2, D5, and D6. (**C**–**F**) Protein analysis of TNF (**C**,**D**), IL-1Ra (**E**,**F**), and IL-1β (**G**,**H**) in microglia cells (pg/mg) and media (pg/mL) D7 after culturing. Statistical data are presented as mean ± SEM (5 experimental cultures/group). * *p* < 0.05, ** *p* < 0.01, *** *p* < 0.001, **** *p* < 0.0001 (**A**–**C**). Scale bar: 20 μm (**A**,**B**), 10 μm (**A**,**B**; GFP^+^ BM cell).

Since TNF and IL-1Ra are clustered, we investigated if changes in TNF expression would also affect the IL-1Ra production by microglia. We harvested microglia from adult tamoxifen-treated *Tnf^fl/fl^* mice and also adult *Cx3cr1^CreER^Tnf^fl/fl^* mice, which displayed a tamoxifen-induced knockdown of TNF in microglia [31]. These cells were cultured and treated with LPS, GFP BM cells, or both, as shown in Figure 5B. First, to confirm the expression of TNF in microglia from tamoxifen-treated *Tnf^fl/fl^* control mice and lack thereof in microglia from *Cx3cr1^CreER^Tnf^fl/fl^* mice, we performed double immunofluorescence stainings. We showed that TNF localized to CD11b^+^ microglia and GFP BM cells in *Tnf^fl/fl^* microglia (Figure 6A), however, exclusively in GFP BM cells in *Cx3cr1^CreER^Tnf^fl/fl^* microglia (Figure 6B).

In vivo tamoxifen treatment of *Cx3cr1^CreER^Tnf^fl/fl^* and *Tnf^fl/fl^* mice prior to microglial isolation and in vitro culturing showed reduced TNF production in microglia and reduced release of TNF into the media of *Cx3cr1^CreER^Tnf^fl/fl^* mice, compared to *Tnf^fl/fl^* controls, by 78% and 94% (Figure 6C). Following LPS stimulation, we observed normal TNF synthesis in microglia derived from *Tnf^fl/fl^* control mice but not in microglia derived from *Cx3cr1^CreER^Tnf^fl/fl^* mice (Figure 6C). In the corresponding medium, we observed an increase in soluble TNF in cultures from *Tnf^fl/fl^* mice, which is a response that was significantly reduced in cultures from *Cx3cr1^CreER^Tnf^fl/fl^* following LPS stimulation (Figure 6D). Following BM and LPS + BM treatment, we observed elevated levels of TNF in microglia and media from both *Tnf^fl/fl^* and *Cx3cr1^CreER^Tnf^fl/fl^* mice, compared to control (Ctl), and LPS stimulated microglia (Figure 6C,D). This demonstrates that GFP BM cells produce and secrete a substantial amount of TNF and that BM cell-mediated TNF production is independent of LPS stimulation.

Next, we tested whether reduced TNF in microglia would affect IL-1Ra expression in microglial cells or media (Figure 6E,F). Generally, the IL-1Ra levels were comparable in both microglia and media from *Tnf^fl/fl^* and *Cx3cr1^CreER^Tnf^fl/fl^* mice, although displaying some within-group variation. IL-1Ra was significantly increased in microglia from *Tnf^fl/fl^*, and *Cx3cr1^CreER^Tnf^fl/fl^* mice stimulated with LPS and treated with BM cells, compared to Ctl (Figure 6E). In the media, following GFP+ BM treatment, IL-1Ra was elevated in cultures from both *Tnf^fl/fl^* and *Cx3cr1^CreER^Tnf^fl/fl^* mice, denoting microglial base-level production of IL-1Ra (Figure 6F). This demonstrated that GFP+ BM cells contributed a significant amount of secreted IL-1Ra, which is an effect that appeared unaffected by LPS stimulation.

Next, we tested whether reduced TNF in microglia would affect IL-1β expression in microglia or media (Figure 6G,H). We generally observed comparable IL-1β levels in microglia and microglial medium derived from *Tnf^fl/fl^* and *Cx3cr1^CreER^Tnf^fl/fl^* mice (Figure 6G). This was independent of the low TNF levels in microglia derived from *Cx3cr1^CreER^Tnf^fl/fl^* mice suggesting some segregation of the molecular pathways for TNF and IL-1β. In the media, we further observed comparable IL-1β levels in microglia originating from *Tnf^fl/fl^* and *Cx3cr1^CreER^Tnf^fl/fl^* mice with the exception of media from microglia treated with LPS + BM, compared to *Tnf^fl/fl^* controls (Figure 6H). This could indicate that other molecular pathways amplified by the absence of TNF in microglia influenced IL-1β secretion by microglia or BM cells.

### 3.6. Toll-Like Receptor 2 Signaling Is Not the Link between TNF and IL-1Ra

Since TNF and IL-1Ra were grouped together based on similarities between genes in the hierarchical clustergram (Figure 5A) and since both colocalized to the same microglial cells in vivo (Figure 5K), we asked whether their expression could be induced by a shared stimulus after tMCAo.

To account for TNF-induced self-expression, we analyzed TNFR1 and TNFR2 expression in LM, LM–LM, and Tg–LM mice 24 h after tMCAo, compared to unlesioned controls, since changes in these receptors could imply autocrine self-induction.

TNFR1 levels were significantly upregulated in LM (mean ± SEM: 5.8 ± 0.8; *p* < 0.001, *n* = 5), LM–LM (5.2 ± 0.6; *p* < 0.01, *n* = 5), and Tg–LM (5.3 ± 0.6; *p* < 0.001, *n* = 5), compared to unlesioned control mice (1.4 ± 0.4; *n* = 5). Additionally, TNFR2 levels were significant upregulated in LM (mean ± SEM: 40.1 ± 5.2; *p* < 0.01, *n* = 4), LM–LM (44.3 ± 4.5; *p* < 0.01, *n* = 4), and Tg–LM (31.8 ± 7.0; *p* < 0.05, *n* = 4), compared to unlesioned control mice (8.6 ± 1.1; *n* = 4). Overall, we observed no difference in TNFR1 and TNFR2 expression between treatment groups poststroke.

**Figure 7:**
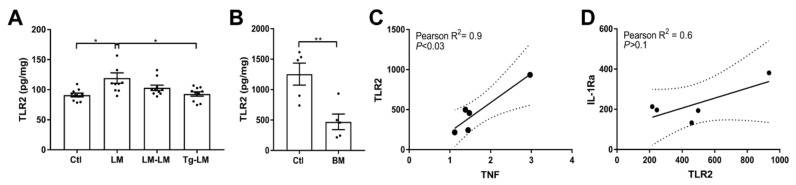
Effect of BM cells on TLR2 protein expression. (**A**) BM cell treatment 30 min after tMCAo in LM–LM and Tg–LM mice, compared to tMCAO LM mice and unlesioned controls (*n* = 10 mice/group). Brain TLR2 protein (pg/mg) was increased 24 h after tMCAo, but this increase was significantly suppressed in IL-1Ra BM treated mice (Tg–LM). (**B**) TLR2 expression (pg/mg) in cultured microglia (Ctl) and BM treated microglial cell cultures. (C, D) Correlation analysis between TLR2 and TNF (**C**) and TLR2 and IL-1Ra (**D**) showed a positive correlation between TLR2 and TNF but not TLR2 and IL-1Ra. Statistical data are presented as mean ± SEM (5 cultures/group). One-way ANOVA with Sidak’s multiple comparison test (**A**), Student’s *t*-test (**B**), and Pearson correlation were performed (**C**,**D**), with significance indicated as * *p* < 0.05, ** *p* < 0.01 (**A**,**B**).

Since knockdown of TNF in adult microglia did not affect IL-1Ra expression, we investigated TLR2, which was identified by STRING analysis (Figure 2C) as a shared receptor likewise involved in the inflammatory defense response. We analyzed TLR2 protein in LM, LM–LM, and Tg–LM mice 24 h after tMCAo. Brain TLR2 protein expression increased 24 h after tMCAo (Figure 7A); however, levels were significantly suppressed following BM treatment with IL-1Ra overproducing cells (Figure 7A). A similar effect on levels of TLR2 was observed in vitro when treating cultured microglia with GFP+ BM cells (Figure 7B).

Correlation analysis was performed to examine for any association between TNF or IL-1Ra and TLR2 in cultured microglia treated with GFP BM cells (Figure 7C). The analysis showed a positive correlation between TNF and TLR2 (Pearson R2 = 0.9, *p* < 0.03) (Figure 7C) but not between IL-1Ra and TLR2 (Pearson R2 = 0.6, *p* > 0.1) (Figure 7D), indicating that TLR2 signaling is not a shared pathway for the induction of TNF and IL-1Ra synthesis in microglia.

## 4. Discussion

The present study shows that TNF, which rises following tMCAo in mice, is significantly impacted by poststroke BM treatment. We show that IL-1Ra-overproducing BM cells, which increase the production of IL-1Ra in microglial cells in situ [11], also enhance the production of TNF and other anti-inflammatory IL-4, and IL-10 in the brain of mice after tMCAo. Studies by others have shown that TNF in synergy with IL-10 and IL-4 can increase the production of IL-1Ra in activated human PMNs in vitro [26]. Here, we show that IL-1Ra producing BM cells increase TNF, IL-10, and IL-4 in the brains of mice 24 h after tMCAo, supporting the view of a regulatory link between these cytokines. The capacity of TNF to induce the production of IL-1Ra by PMNs has previously been described [38,39]; however, we found that the production of IL-1Ra in microglial cells was TNF independent, as demonstrated in cultured microglia from mice with Tamoxifen-induced knockdown of TNF. Both IL-4 and IL-10 are considered potent anti-inflammatory cytokines, and systemic administration of both has been shown to improve recovery poststroke [40,41,42,43]. Low IL-4 has been shown to enhance excitatory transmission, thereby aggravating cerebral ischemia [42], and low IL-10 has been shown to exacerbate the inflammatory response after MCAo [41]. A BM-mediated increase in both IL-4 and IL-10 may therefore imply a more favorable microenvironment in the brain poststroke.

As noted previously, the IL-1Ra-overproducing BM cells reduced IL-1β mRNA and protein levels in the brain 24 h after MCAo [11], including CXCL1 and IL-12p70 levels, as shown in this study. CXCL1 is a known neutrophil chemoattractant [44] and inducer of IL-12p70 [45], and it has been suggested that the central-to-the-peripheral gradient of CXCL1 is crucial [46] for neutrophil recruitment after acute injury [47]. High CXCL1 levels measured in the cerebrospinal fluid of stroke patients have been shown to correlate with larger infarct volumes [48], supporting the view that CXCL1 plays a critical role in the pathophysiology of stroke. It has also been reported that patients with high neutrophil activity in the brain have elevated IL-12p70 levels [49] and that IL-12p70 plays a detrimental role in stroke [50]. These findings support our view that reduced levels of CXCL1 and IL-12p70 are a beneficial effect of BM cell treatment. Other proteins such as IFNγ, IL-2, IL-5, and IL-6 were not notably affected by the presence of BM cells in the brain, and our study shows that BM cells do not affect the levels of TNF, IL-4, IL-10, CXCL1, IL12p70, IFNγ, IL-1, IL-5, or IL-6 in serum 24 h after tMCAo in mice.

BM cells are believed to modulate a number of very diverse effects, ascribed both to the secretion of anti-inflammatory and neurotrophic factors and to direct cellular cross talk [51,52]. Gene expression changes have been studied poststroke using microarray analysis; however, only a few studies were ever validated by qPCR [53]. Here, we show by qPCR that BM cells, which have infiltrated the neural parenchyma [11], significantly downregulate a panel of harmful stroke-related mRNAs such as *Arg*, a marker of poor functional outcome and stroke severity [54], *Cxcr2*, which plays a role in immune cell trafficking during inflammation [55,56], *Fpr1*, which is considered responsible for attenuation of inflammation and neutrophil activation during inflammation [57,58], *Il1r2* a known decoy receptor and regulator of IL-1α activity [59], *S100a8* and *S100a9*, which are passively released by necrotic cells or from activated immune cells [60,61,62], and *Slpi*, a ubiquitous serine protease inhibitor produced by both neutrophils and macrophages [63], overall underscoring BM cells as anti-inflammatory, with a regulatory effect on immune cell infiltration.

In this study, hierarchical cluster analysis showed an association between *Tnf* and *Il1rn* mRNA in the brains of mice subjected to tMCAo and tMCAo + BM treatment, and when investigating the ability of BM cells to shape microglial cytokine production in vitro, we could also confirm a positive correlation between TNF and IL-1Ra protein in microglia and media. By 24 h, we likewise observed TNF and IL-1Ra coproducing microglia in the ischemic penumbra of mice, supporting the view that TNF and IL-1Ra production could be linked in distinct subsets of microglial cells poststroke. TNF expression can be self-induced via the classical nuclear factor kappa B (NFκB) pathway typically activated via TNFR1 or TNFR2 [64]. However, we observed no effect of IL-1Ra producing BM cells on TNFR1 or TNFR2 levels 24 h after tMCAo mice, suggesting an alternative pathway to be involved in the induction of TNF and IL-1Ra in mice after tMCAo.

Poststroke, TLRs are recognized as an immune communicatory link between the CNS and the periphery [65,66]. Considering that BM-derived mesenchymal stem cells (MSCs) dynamically express a number of Toll-like receptors such as TLR1, TLR2, TLR3, TLR4, TLR5, and TLR6 [67,68], which in parallel also tailors the innate immune response in microglia [69], TLRs could be involved in the cellular communication between BM cells and microglia. Their extracellular domains are known to mediate Toll-to-Toll contact across different cell lineage [70] and linking the innate and adaptive immune response in vivo [71,72]. Our results support findings of increased TLR2 expression following tMCAo in mice [73] and findings in rats subjected to multiple trauma, reporting on downregulation of TLR2 expression after BM treatment [74]. Although TLR2 expression correlated with TNF expression, it did not correlate with IL-1Ra expression, arguing against TLR2 signaling as an amplifier of TNF and IL-1Ra in cultured microglia treated with BM cells. However, TLR signaling does not operate in isolation in vivo but in synergy with other receptors such as TNFR1 [75] and the IL-1 receptor type 1 [75,76].

Besides microglia, there appears to be cross talk between BM-derived MSCs and macrophages [77] and/or neutrophils [78]. MSCs, a recognized source of IL-1Ra [79], have been proposed to induce a paracrine-like reprogramming of inflammatory macrophages into an anti-inflammatory phenotype [77,80], increasing the levels of IL-10 while lowering IL-1β and IL-17 [81]. Similarly, IL-6 derived from MSCs has been shown to protect neutrophils from apoptosis, while IL-1Ra inhibits TNF production by activated macrophages [82]. The reprograming relationship between BM-derived MSCs and macrophage/neutrophils is particularly interesting since these cells are the first to arrive at the infarcted brain poststroke [8,10,11], potentially enhancing a more protective and regenerative microenvironment [83,84]. Our results support the view that the immunosuppressive capacity of BM-derived MSCs is not innate but most likely induced under inflammatory conditions in situ [85]. In our view, there is no doubt that BM-derived MSCs have a beneficial effect in stroke as reviewed for both rodent and human stroke trials [1,65,86,87,88]; however, further details are needed in order to understand the mechanism of these cells poststroke fully.

## 5. Conclusions

In summary, this study shows that BM cells with a high expression of IL-1Ra increase TNF, IL-10, and IL-4 levels while decreasing CXCL1, IL-12p70, and TLR2 levels poststroke. Based on our hierarchical cluster analysis and correlation analysis, we suggest a strong link between TNF and IL-1Ra, which are expressed largely by the same subset of microglia cells poststroke. Combined with knowledge of decreased IL-1β production, smaller infarct volumes, and improved functional outcome, this information helps to understand how BM cells promote neuroprotection after tMCAo in mice, a potential future stroke therapy.

## Data Availability

Request to access datasets should be directed to bclausen@health.sdu.dk.

## Supplementary Materials

The following are available online at https://www.mdpi.com/article/10.3390/cells10040956/s1, Supplemental Figure S1. Gene expression affected by post-stroke BM treatment in mice. (A,B) Scatter and Volcano plots showing gene changes in LM-LM treated (*n* = 5 mice) (A) and Tg-LM treated mice (*n* = 5 mice) (B) 24 hours after tMCAo compared to unlesioned control mice (*n* = 11). Table S1: Genes identified by comparison to Ctl and tMCAo, followed by BM treatment. All genes were identified by qPCR, upregulated gene transcripts beyond a Log2 Fc are depicted in red color, and downregulated transcripts in green color.
