# Supplementary material for: Bone Marrow-Derived IL-1Ra Increases TNF Levels Poststroke
Source: Cells. 2021 Apr 20;10(4):956. doi: 10.3390/cells10040956 (PMC8074385; doi:10.3390/cells10040956)
Supplement: Supplementary file 1 [file cells-10-00956-s001.zip › cells-1143971-supplementary.pdf]

cells

MDPI

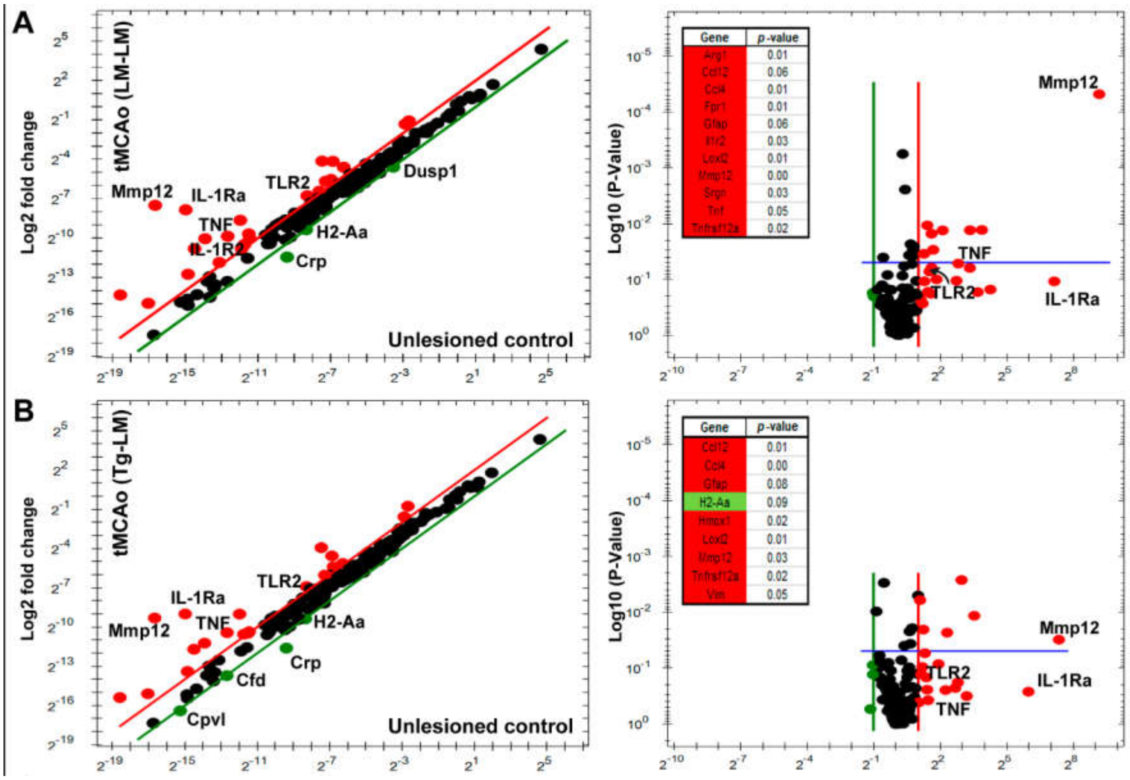

Supplementary Figure S1. Gene expression affected by post-stroke BM treatment in mice. (**A**-**B**) Scatter and Volcano plots showing gene changes in LM-LM treated (n = 5 mice). (**A**) and Tg-LM treated mice (n = 5 mice). (**B**) 24 hours after tMCAo compared to unlesioned control mice (n = 11 mice).

Supplementary Table 1. Genes changes identified by comparison.

Upregulated genes
Downregulated genes

**Ctl vs.**			**tMCAo vs.**		**tMCAo (LM-LM) vs.**
tMCAo	**tMCAo (LM-LM)**	**tMCAo (Tg-LM)**	**tMCAo (LM-LM)**	**tMCAo (Tg-LM)**	**tMCAo (Tg-LM)**
Ahnak	Arg1	Arg1	Arg1	Arg1	Arg1
Anxa1	Ccl12	Ccl12	Cpvl	Btg1	Cpvl
Arg1	Ccl4	Ccl4	Cxcr2	Ccr1	Cxcr2
Ccl12	Ccr1	Ccr1	Fabp4	Ccr7	Hp
Ccl4	Crp	Cfd	Fpr1	Cfd	Il1rn
Ccr1	Cxcr2	Cpvl	Il1r2	Cxcr2	Mmp12
Cd163	Dusp1	Crp	S100a8	Fabp4	
Cd36	Fabp4	Cxcr2	S100a9	Fpr1	
Crp	Fpr1	Fabp4	Slpi	Hp	
Cxcr2	Gfap	Fkbp5		Ier3	
Cyp1b1	H2-Aa	Fpr1		Il1r2	
Dusp1	Hmox1	Gfap		Il1rn	
Fabp4	Hp	H2-Aa		Mmp12	
Fkbp5	Il1r2	Hmox1		S100a8	
Fpr1	Il1rn	Il1r2		S100a9	
Gfap	Loxl2	Il1rn		Slpi	
Ghrl	Mmp12	Loxl2		Srgn	
Hmox1	Plin2	Mmp12		Tnfrsf12a	
Hp	Slpi	Srgn			
Ier3	Srgn	Tlr2			
Il13ra1	Tlr2	Tnf			
Il1r2	Tnc	Tnfrsf12a			
Il1rn	Tnf	Vim			
Loxl2	Tnfrsf12a	Vsig4			
Mmp12	Vim				
Plin2	Vsig4				
S100a8					
S100a9					
Slc6a4					
Slpi					
Srgn					
Tlr2					
Tlr7					
Tnc					

Tnf<br>Tnfrsf12a<br>Vim<br>Vsig4					
